# Enhanced dielectric properties of poly(vinylidene fluoride) composites filled with nano iron oxide-deposited barium titanate hybrid particles

**DOI:** 10.1038/srep33508

**Published:** 2016-09-16

**Authors:** Changhai Zhang, Qingguo Chi, Jiufeng Dong, Yang Cui, Xuan Wang, Lizhu Liu, Qingquan Lei

**Affiliations:** 1 Key Laboratory of Engineering Dielectrics and Its Application, Ministry of Education, Harbin University of Science and Technology, Harbin 150080, P. R. China; 2 School of Materials Science and Engineering, Harbin University of Science and Technology, Harbin 150080, P. R. China; 3 School of Applied Science, Harbin University of Science and Technology, Harbin 150080, P. R. China; 4 State Key Laboratory of Electrical Insulation and Power Equipment, Xi’an Jiaotong University, Xi’an 710049, P. R. China

## Abstract

We report enhancement of the dielectric permittivity of poly(vinylidene fluoride) (PVDF) generated by depositing magnetic iron oxide (Fe_3_O_4_) nanoparticles on the surface of barium titanate (BT) to fabricate BT–Fe_3_O_4_/PVDF composites. This process introduced an external magnetic field and the influences of external magnetic field on dielectric properties of composites were investigated systematically. The composites subjected to magnetic field treatment for 30 min at 60 °C exhibited the largest dielectric permittivity (385 at 100 Hz) when the BT–Fe_3_O_4_ concentration is approximately 33 vol.%. The BT–Fe_3_O_4_ suppressed the formation of a conducting path in the composite and induced low dielectric loss (0.3) and low conductivity (4.12 × 10^−9^ S/cm) in the composite. Series-parallel model suggested that the enhanced dielectric permittivity of BT–Fe_3_O_4_/PVDF composites should arise from the ultrahigh permittivity of BT–Fe_3_O_4_ hybrid particles. However, the experimental results of the BT–Fe_3_O_4_/PVDF composites treated by magnetic field agree with percolation theory, which indicates that the enhanced dielectric properties of the BT–Fe_3_O_4_/PVDF composites originate from the interfacial polarization induced by the external magnetic field. This work provides a simple and effective way for preparing nanocomposites with enhanced dielectric properties for use in the electronics industry.

Dielectric materials that possess high dielectric permittivity (*ε*) and dielectric field strength without excessive dielectric loss are necessary to meet the miniaturization requirements of microelectronic device-structures, including gate dielectrics, high charge-storage capacitors and electro-active materials^1^,^2^,^3^. Polymer materials are currently of considerable interest as high-permittivity materials for electronics applications. However, their low dielectric permittivity limits their application. Many strategies to increase the dielectric permittivity of polymers have been reported^4^ and the introduction of high dielectric permittivity nanoparticles (e.g., CaCu_3_Ti_4_O_12_ (CCTO) and BT nanoparticles) into a polymer matrix has been widely adopted^5^,^6^,^7^,^8^,^9^. Unfortunately, the dielectric permittivity improvement for two-phase composite materials is still limited and often requires a large filler loading (>60 vol.%) to enhance the dielectric permittivity, which causes the materials to lose their flexibility and uniformity. Investigations of percolative materials have been carried out by incorporating metal powders or other conductive fillers into a polymer matrix^10^. Ultra-high dielectric constant values can be achieved; however, a high conductivity and dielectric loss also result when the filler content approaches the percolation threshold.

To overcome these limitations, researchers have focused on improving the dielectric properties of the materials *via* surfactant treatment of the filler by a coupling agent^10^,^11^,^12^,^13^,^14^. Improving the physical compatibility at a novel interface can guarantee good dispersion of the ceramic particles. Luo *et al*.^12^ modified BT nanoparticles using hydantoin epoxy resin and found that hydantoin/BT–P(VDF-HFP) (P(VDF-HFP): poly(vinylidene fluoride-*co*-hexafluoropropylene) nanocomposites had a high dielectric permittivity (*ε* = 48.9) and a low dielectric loss (0.06) with 50 vol.% filler loading at 1 kHz. Fu *et al*.^13^ modified BT particles using polyvinyl pyrrolidone (PVP) fillers to realize composites with high dielectric permittivity (*ε* ≈ 120) and low loss tangent (tan *δ* ≈ 0.3) with 60 vol.% filler loading at 100 Hz. Another promising strategy is to fabricate three-phase polymeric composites containing conductive fillers^15^,^16^. Yang and co-workers^15^ prepared Ni/CCTO/PVDF composites with a dielectric constant (140) and a dielectric loss of 0.5 near the percolation threshold when the filler content of Ni and CCTO was 60 vol.%. Many researchers have reported that nano-sized Ag particles discretely deposited on the surface of the ceramic can efficiently enhance the dielectric permittivity of the composites^17^,^18^,^19^. Luo *et al*.^17^ prepared PVDF embedded with BT–Ag nanoparticles and found that the BT–Ag/PVDF composites with 56.8 vol.% filler loading presented a high dielectric permittivity (*ε* = 160) and a low dielectric loss (0.11) at 1 kHz. However, even with the high-volume fraction of inorganic compounds in the composite, the dielectric permittivity of the composite was not high enough for the practical application.

Shear flow, magnetic field, electric field, or electric force can change the molecular arrangement of a polymer and the distribution of conductive particles in a host polymer, which influence the microstructure and macro-properties of composites^20^,^21^,^22^,^23^,^24^. In this study, we fabricated super-paramagnetic Fe_3_O_4_ nanoparticles deposited on the surface of BT ceramic particles via a chemical precipitation method. On this basis, we have designed BT–Fe_3_O_4_/PVDF composites treated under a constant magnetic field for 30 min at 60 °C. The morphology of BT–Fe_3_O_4_ particles was characterized by transmission electron microscopy (TEM), and the composites were studied by scanning electron microscopy (SEM). The effect of an external magnetic field on the dielectric properties of the composites filled by BT–Fe_3_O_4_ and correlation with the structure and morphology of the composites are also discussed systematically.

## Results and Discussion

### Characterization of the BT–Fe_3_O_4_ hybrid particles and BT–Fe_3_O_4_/PVDF composites

**Figure 1:**
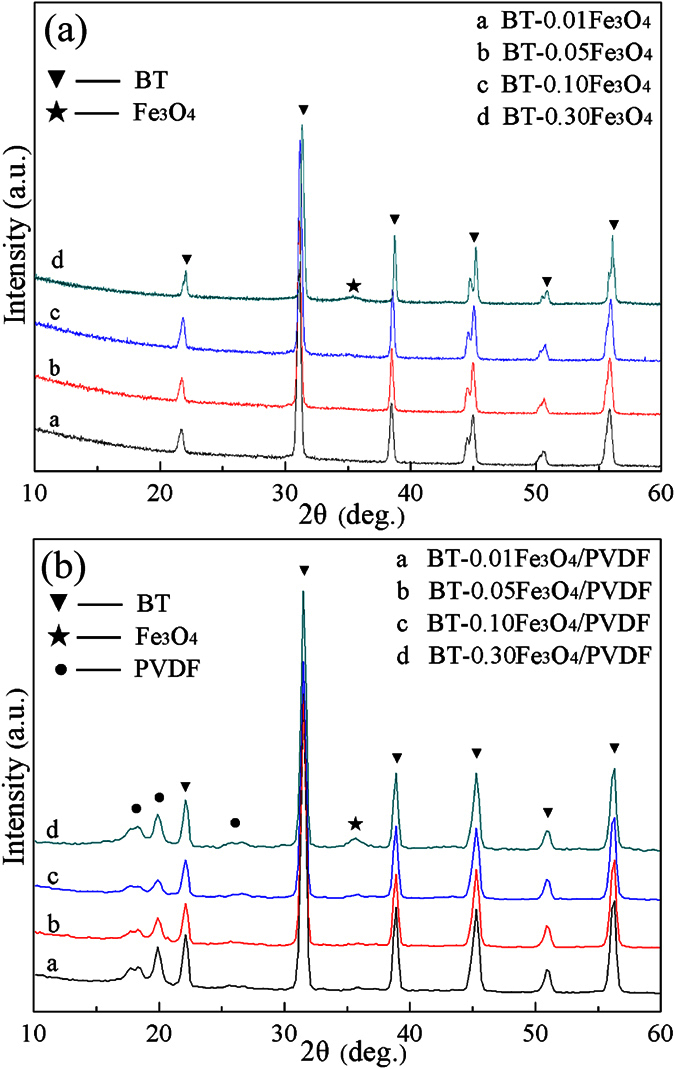
(**a**) XRD patterns from BT–Fe_3_O_4_ hybrid particles with different BT and Fe_3_O_4_ ratios. (**b**) XRD patterns from BT–*x*Fe_3_O_4_/PVDF composites with 20 vol.% BT–*x*Fe_3_O_4_.

Figure 1a shows XRD patterns from BT–Fe_3_O_4_ hybrid particles with different the volume fraction of Fe_3_O_4_. The characteristic diffraction peaks of BT appear at 2θ = 22°, 31°, 38°, 45° and 56° corresponding to the diffraction peaks from (010), (110), (111), (200) (002), and (211), respectively. When the diffraction angle was 45°, splitting of the (200) and (002) peaks was observed, which indicates that the BT nanoparticles had a tetragonal phase structure. Diffraction peaks characteristic of Fe_3_O_4_ were observed in the patterns from the BT−*x*Fe_3_O_4_ hybrid particles when the volume fraction of Fe_3_O_4_ increased to 30 vol.%. Figure 1b shows that the diffraction peak of Fe_3_O_4_ appeared at 2θ = 35.6° for the BT−*x*Fe_3_O_4_/PVDF composites. The XRD patterns of the composites clearly demonstrated that the BT–Fe_3_O_4_ hybrid particles filled the polymer matrix.

**Figure 2:**
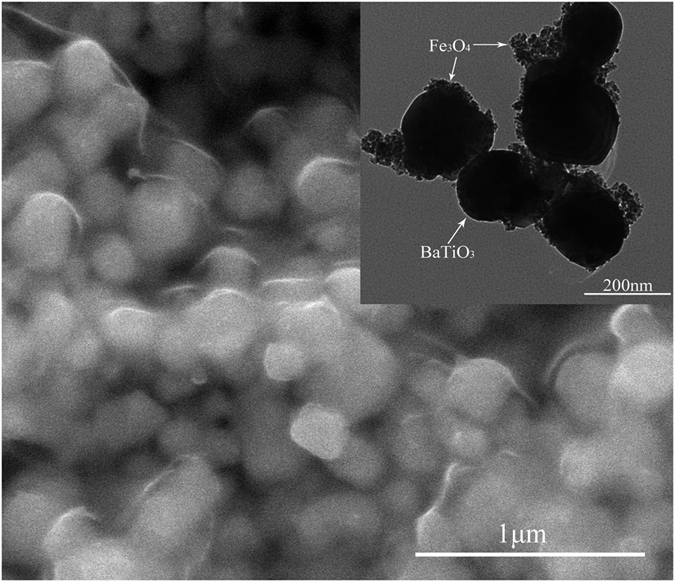
SEM image of BT–Fe_3_O_4_ hybrid particles. The inset shows the TEM image of BT–Fe_3_O_4_ hybrid particles.

**Figure 3:**
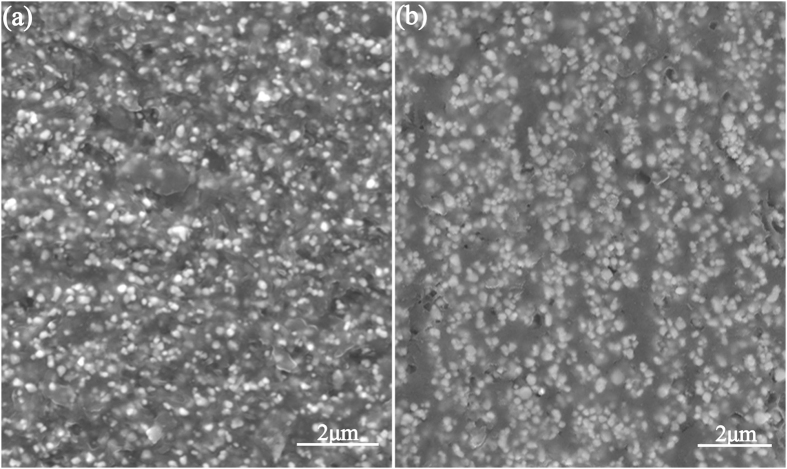
(**a**) Cross-sectional SEM image of a fractured BT–Fe_3_O_4_/PVDF composite with 33 vol.% BT–Fe_3_O_4_ filler. (**b**) Cross-sectional SEM image of a fractured BT–Fe_3_O_4_/PVDF composite with 33 vol.% BT–Fe_3_O_4_ filler annealed under a magnetic field.

SEM and TEM were used to study the size and morphology of the BT–Fe_3_O_4_ nanoparticles. The BT particles were spherical in shape and had an average diameter of approximately 200 nm, as shown in Fig. 2. The TEM image of the BT–Fe_3_O_4_ hybrid particles, shown in the inset of Fig. 2, showed that the Fe_3_O_4_ nanoparticles were discontinuously deposited on the surface of the BT, and the Fe_3_O_4_ nanoparticles were significantly smaller than the BT particles (diameters of 10–20 nm). There was some agglomeration of the Fe_3_O_4_ nanoparticles on the surface of the BT ceramic powders. This phenomenon occurs because the Fe_3_O_4_ nanoparticles have a high specific surface area and are prone to agglomeration. Figure 3a depicts a SEM image of a fractured cross-section of BT–Fe_3_O_4_/PVDF composite with 33 vol.% BT–Fe_3_O_4_ filler. It can be found that the BT–Fe_3_O_4_ hybrid particles are homogeneously embedded in the PVDF matrix to form a random composites without obvious agglomeration. Figure 3b shows a cross-sectional SEM image of BT–Fe_3_O_4_/PVDF# composites with 30 vol.% BT–Fe_3_O_4_, some of the magnetic BT–Fe_3_O_4_ hybrid particles showed a directional arrangement along the direction of the magnetic field when the BT–Fe_3_O_4_/PVDF composites were treated under a constant magnetic field for 30 min at 60 °C; although some BT–Fe_3_O_4_ hybrid particles formed clusters, it is impossible for them to form a conductive network through the whole system. This phenomenon also indicated that magnetic fields have changed the distribution of the BT–Fe_3_O_4_ hybrid particles in the PVDF matrix.

### Dielectric properties of the BT–Fe3O4/PVDF composites

**Figure 4:**
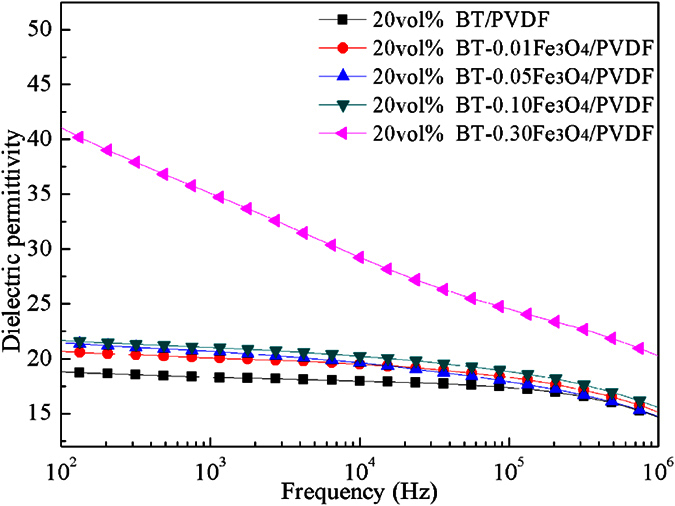
Dependence of dielectric permittivity on frequency of the 20 vol.% BT–*x*Fe_3_O_4_/PVDF composites at room temperature.

To obtain the composite materials with excellent dielectric properties, the effects of different Fe_3_O_4_ volume fractions in the BT–Fe_3_O_4_ particles on the electrical properties were explored. The dependence of dielectric permittivity on the frequency of the 20 vol.% BT–*x*Fe_3_O_4_/PVDF composites in the frequency range from 100 Hz to 1 MHz at room temperature is shown in Fig. 4. The dielectric permittivity of the composites showed a weak frequency dependence when the volume fraction of Fe_3_O_4_ was less than 10 vol.%. When the volume fraction of Fe_3_O_4_ was greater than 10 vol.%, the dielectric permittivity increased significantly and the frequency dependence of the dielectric permittivity of composites gradually increased as the volume fraction increased, especially at low frequency. The dielectric permittivity of the 20 vol.% BT–*x*Fe_3_O_4_/PVDF composite was 42 when the volume fraction of Fe_3_O_4_ reached 30 vol.%, which is 1.2 times higher than that of 20 vol.% BT/PVDF. This demonstrates that incorporating conducting fillers into the polymer matrix results in an increase in dielectric permittivity. The increased conductivity of the interlayer between the BT and PVDF matrix created by the Fe_3_O_4_ enhances the space charge polarization and Maxwell–Wagner–Sillars effect, which play an important role in improving the dielectric permittivity^25^,^26^.

**Figure 5:**
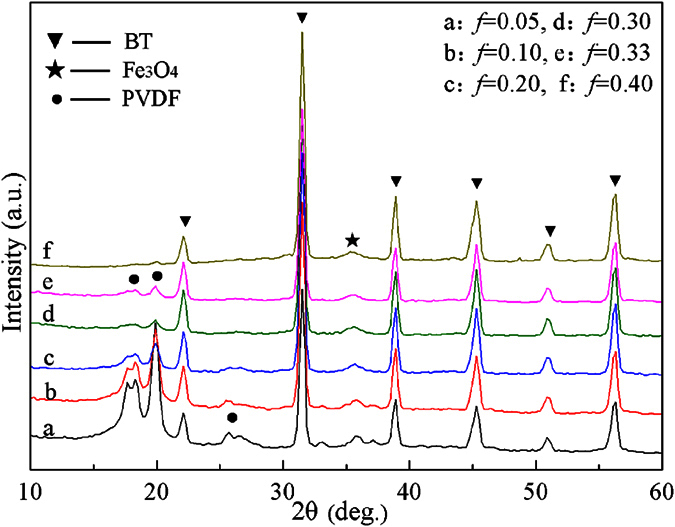
XRD patterns from the BT–0.3Fe_3_O_4_/PVDF composites with BT–0.3Fe_3_O_4_ volume fractions between 5 vol.% and 40 vol.%.

To understand the influence of the BT–Fe_3_O_4_ hybrid particles, the XRD patterns of BT–0.3Fe_3_O_4_/PVDF composites with BT–0.3Fe_3_O_4_ volume fractions between 5 vol.% and 40 vol.% are shown in Fig. 5. In these patterns, the BT peaks did not show reflection splitting and no super-lattice reflections or secondary phases were present. After the BT–0.3Fe_3_O_4_ filler was incorporated into the PVDF matrix, the intensities of PVDF peaks were reduced and the BT and Fe_3_O_4_ peaks became sharper and stronger. When the volume fraction of the BT–0.3Fe_3_O_4_ filler increased to 40 vol.%, the PVDF peaks became very weak because of the strong diffraction from the incorporated ceramic powders.

**Figure 6:**
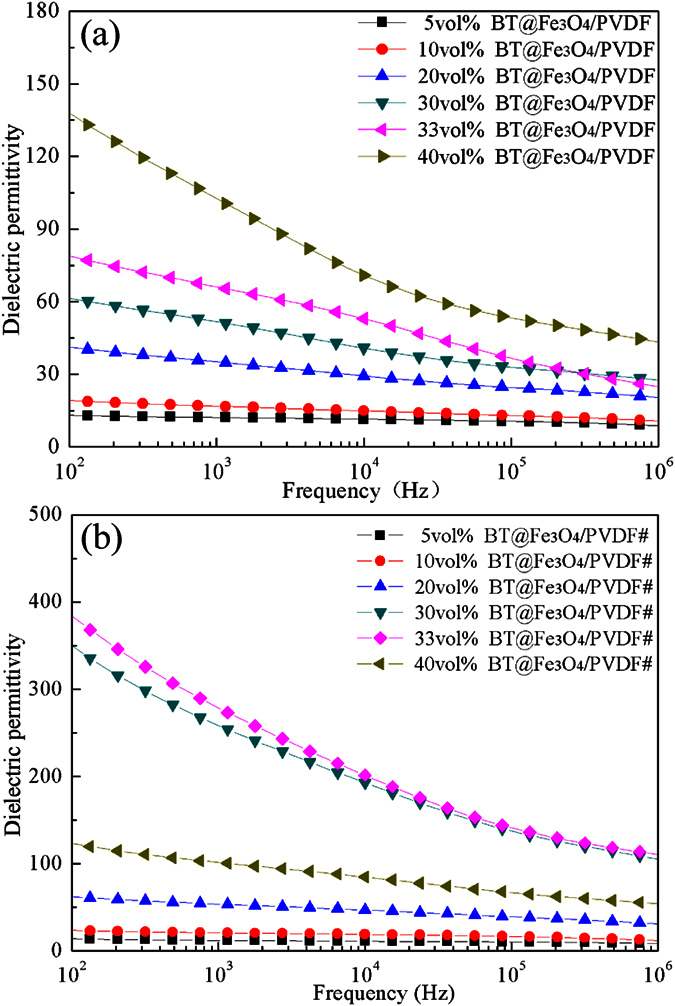
Dependence of dielectric permittivity on frequency of the (**a**) BT–Fe_3_O_4_/PVDF composites and (**b**) BT–Fe_3_O_4_/PVDF# composites at room temperature.

Based on the above-mentioned results, the BT–0.3Fe_3_O_4_/PVDF composites were studied systematically at the following research. The dependence of the dielectric permittivity of the BT–Fe_3_O_4_/PVDF composites with the different volume fractions of BT–Fe_3_O_4_ at room temperature is shown in Fig. 6a. The dielectric permittivity increased significantly when the volume fraction of BT–Fe_3_O_4_ increased. At 40 vol.% BT–Fe_3_O_4_ hybrid particles, the dielectric permittivity was 138, which is 15 times higher than that of the pure PVDF matrix. However, the dielectric permittivity of the composites was still not high enough for embedded devices. On this basis, we designed BT–Fe_3_O_4_/PVDF composites by applying an external magnetic field. The frequency dependence of the dielectric properties of the BT–Fe_3_O_4_/PVDF# composites is shown in Fig. 6b. Dielectric permittivity increases with volume fraction up to 33 vol.%, and then decreases when volume fraction exceeds 33 vol.%. The dielectric permittivity of the BT–Fe_3_O_4_/PVDF# composites reached 385, which is 1.8 times higher than that of the 40 vol.% BT–Fe_3_O_4_/PVDF composites and this value is higher than that of many previous reports^12^,^17^,^27^,^28^,^29^,^30^,^31^,^32^. For example, as shown in Table 1, this value was found to be significant larger than that of BT@SnO_2_/PVDF composites containing 45vol.% BT@SnO_2_ (≈160)^29^. It should be noted that a high dielectric permittivity (280) of BT–Fe_3_O_4_/PVDF# composites was obtained at 1 kHz and this value is superior to that of BT–Ag/PVDF composites with higher filler loading. In that report, the highest dielectric permittivity reported by Luo *et al*.^17^ for 56.8 vol.% BT–Ag hybrid particles filled into PVDF was 160 at 1 kHz. Moreover, the amount of filler in the BT–Fe_3_O_4_/PVDF# composites was smaller than that in other materials described in the literature, and displayed better flexibility. In addition, compared with BT–Fe_3_O_4_/PVDF composites, the dielectric permittivity of the BT–Fe_3_O_4_/PVDF# composites was increased greatly, and this result also indicated that the applied magnetic field can greatly affect the dielectric properties of the BT–Fe_3_O_4_/PVDF composites.

**Figure 7:**
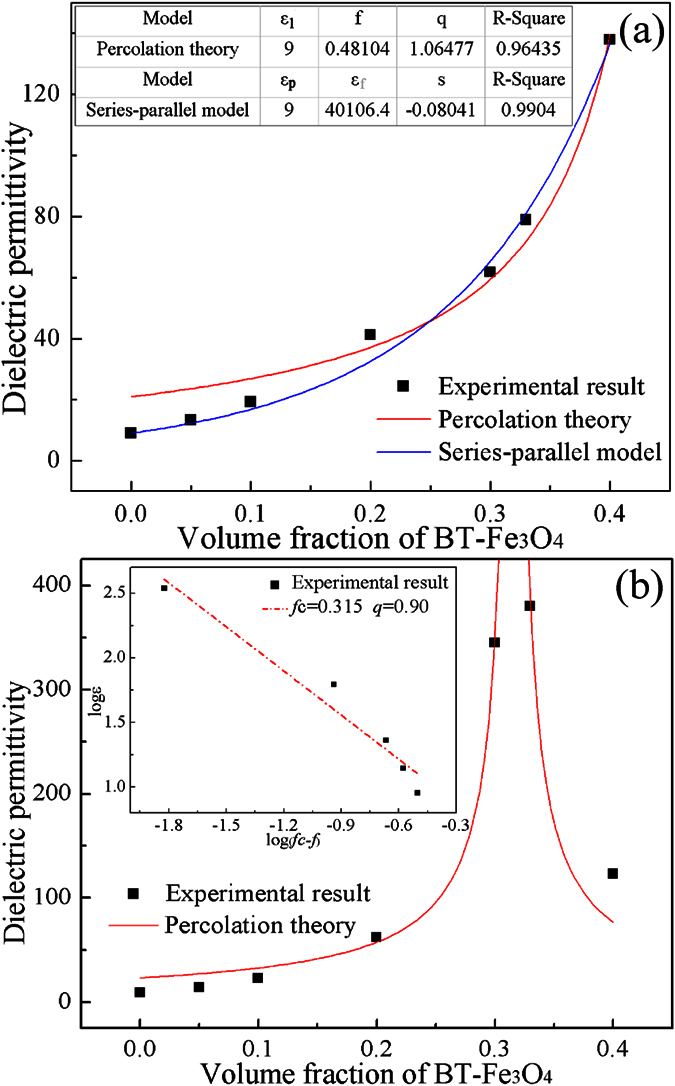
Experimental and theoretical dielectric permittivity of the (**a**) BT–Fe_3_O_4_/PVDF and (**b**) BT–Fe_3_O_4_/PVDF# composites with various volume fractions of BT–Fe_3_O_4_ at 100 Hz and room temperature.

The structure of the BT–Fe_3_O_4_ hybrid particles means that we can regard each particle as a unit. The classic percolation theory was used to predict the dielectric behavior of the BT–Fe_3_O_4_/PVDF composites^33^. The dielectric behavior of the BT–Fe_3_O_4_/PVDF composites yields to the classic percolation theory as below:


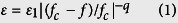


where *ε* and *ε*_1_ are the dielectric permittivity of the composites and PVDF matrix, respectively, *f* is the volume fraction of BT–Fe_3_O_4_ and *f*_c_ is the percolation threshold, *q* is a critical exponent. As shown in Fig. 7a, the experimental results agreed well with the percolation theory when the volume fraction of the filler was greater than 10 vol.%. However, for volume fractions less than 10 vol.%, the fitting results deviate from the experimental data apparently, indicating that the BT–Fe_3_O_4_ hybrid particle is not a real conducting phase in the composites. In this study, a series-parallel model was employed to estimate the permittivity of the BT–Fe_3_O_4_ hybrid particles^4^,^18^.


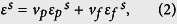


here *ε, ε*_*p*_, and *ε*_*f*_ are dielectric permittivity of the composites, PVDF matrix, and the BT–Fe_3_O_4_ hybrid particles, respectively, v_*p*_ and v_*f*_ are the volume fractions of the PVDF matrix and the BT–Fe_3_O_4_ hybrid particles, and the parameter *s* is the depolarization factor. As shown in Fig. 7a, the experimental results for the BT–Fe_3_O_4_/PVDF composites fit well with the series-parallel model. Moreover, we can find that the dielectric permittivity of BT–Fe_3_O_4_ hybrid particles is 40106 at 100 Hz, which is over 10 times higher than that of the pure BT (*ε* = 3000). That is, the high dielectric permittivity of BT–Fe_3_O_4_/PVDF composites is mainly attributed to the huge dielectric permittivity of BT–Fe_3_O_4_ hybrid particles.

However, for the BT–Fe_3_O_4_/PVDF# composites, it can be found that the percolation theory agrees well with the experimental results, though the fitting results also deviate from the experimental data at low volume fraction of BT–Fe_3_O_4_ loading (see Fig. 7b). The fitting parameters *f*_*c*_ and *q* are 31.5 vol.% and 0.90, respectively. The linear fit of the log value of the dielectric permittivity and volume fraction also indicates that the dielectric permittivity fits well with percolation theory (see Fig. 7b inset). The inter-particle distance would decrease as the volume fraction of the BT–Fe_3_O_4_ hybrid particles increased, and that the probability of BT–Fe_3_O_4_ hybrid particles coming into contact increased because of the high-intensity magnetic field (see Fig. 3b). Fe_3_O_4_ with high conductivity can produce electrical current under an applied filed and the charges will move and accumulate at the interface between the Fe_3_O_4_ and PVDF matrix. The charge accumulation will result in enhanced polarization and dielectric response under the electric filed. That is, the percolation effect was induced by the external magnetic field, which could effectively enhance the interfacial polarization of the BT–Fe_3_O_4_/PVDF composites.

**Figure 8:**
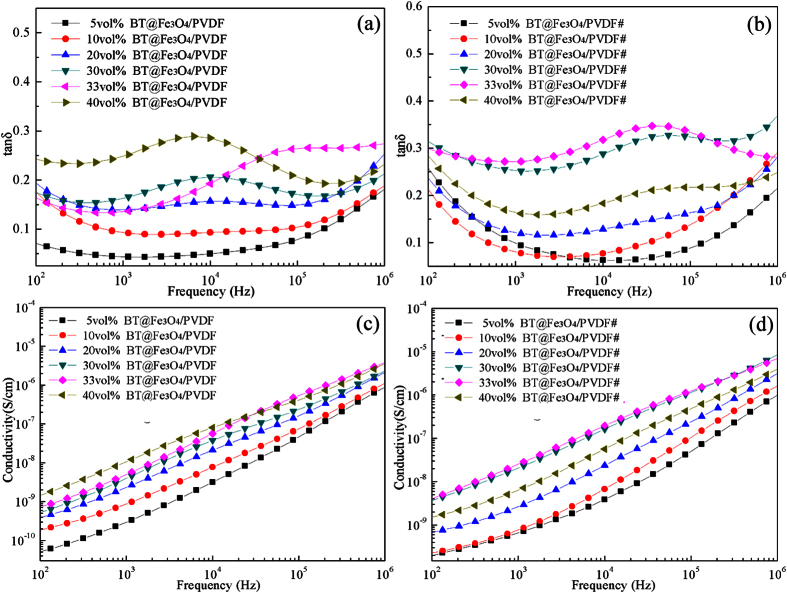
Dependence of dielectric loss of the (**a**) BT–Fe_3_O_4_/PVDF composites and (**b**) BT–Fe_3_O_4_/PVDF# composites on frequency at room temperature. Dependence of conductivity of the (**c**) BT–Fe_3_O_4_/PVDF composites and (**d**) BT–Fe_3_O_4_/PVDF# composites on frequency at room temperature.

The energy loss due to the consumption of a dielectric material can be determined by the following equation:


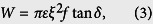


where *ξ* is the electric field strength and *f* is the frequency. For embedded capacitor applications, the dielectric loss is an essential parameter. The dielectric loss measured at a given frequency includes polarization loss and conduction loss^19^. The loss tangent as a function of frequency for the BT–Fe_3_O_4_/PVDF composites is shown in Fig. 8a. It can be found that the dielectric loss remained low (tan*δ* < 0.3) over the whole frequency range. The conduction loss is caused by charge flow through the composites, which depends on the electric conductivity of the composites. As shown in Fig. 8c, the conductivity of the composites with a filler loading of 5 vol.% remained low (5 × 10^−11^ S/cm) because the absorbed insulating polymer chains act as the dielectric barrier governing the tunneling conduction and make it impossible for complete contact between the nanoparticle clusters^34^,^35^. The conductivity of the BT–Fe_3_O_4_/PVDF composites increased as the BT–Fe_3_O_4_ loading increased. The conductivity increased from 5 × 10^−11^ S/cm to 1.4 × 10^−9^ S/cm at 100 Hz, indicating that a conducting path was not formed in the composites, in agreement with the low dielectric loss (shown in Fig. 8a). As shown in Fig. 8b,d, a relatively low dielectric loss (0.3) and a low conductivity (4.12 × 10^−9^ S/cm) were obtained when the volume fraction of BT–Fe_3_O_4_ was 33 vol.%. Compared with the BT–Fe_3_O_4_/PVDF composites, the BT–Fe_3_O_4_/PVDF# composites exhibited a substantial increase of dielectric permittivity, a slight increase of dielectric loss as well as a slight increase of conductivity. In general, percolative composites can exhibit very high dielectric constants at the proper filler loading. However, these composites also exhibit a relatively high conductivity due to the insulator–conductor transition near the percolation threshold. In the present study, the insulating BT particles lower the probability of the Fe_3_O_4_ particles coming into contact because they are discontinuous and discretely fixed on the BT surface. The BT–Fe_3_O_4_ hybrid particles made it difficult for the Fe_3_O_4_ particles to form a complete conductive network throughout the whole system, resulting in composites with high dielectric permittivity, low dielectric loss, and low conductivity.

## Conclusion

In summary, BT–Fe_3_O_4_/PVDF composites with high dielectric permittivity, low dielectric loss, and low conductivity were obtained by annealing under an external magnetic field. TEM images showed that Fe_3_O_4_ nanoparticles with an average size of 10–20 nm were discontinuously and discretely deposited on the BT surface. The magnetic field made the BT–Fe_3_O_4_ particles move in the PVDF matrix and enhanced the probability of forming BT–Fe_3_O_4_ clusters. The structure of the BT–Fe_3_O_4_ suppressed the formation of a conducting path in the composites. The dielectric permittivity of the BT–Fe_3_O_4_/PVDF composites increased following annealing under a magnetic field for 30 min at 60 °C, but the dielectric loss and conductivity remained low. The experimental results for the BT–Fe_3_O_4_/PVDF composites fit well with the series-parallel model, indicating that the enhanced dielectric permittivity of BT–Fe_3_O_4_/PVDF composites is mainly attributed to the ultrahigh dielectric permittivity of BT–Fe_3_O_4_ hybrid particles. However, the percolation effect was induced by the external magnetic field, which could effectively enhance the interfacial polarization of the BT–Fe_3_O_4_/PVDF# composites. The high dielectric permittivity, low dielectric loss, and low conductivity of these composites make them suitable candidates for use in embedded devices in the electronics industry.

## Material and Methods

### Preparation of BT–Fe_3_O_4_ hybrid particles

BT–Fe_3_O_4_ hybrid nanoparticles were prepared *via* a chemical precipitation method. 35 g of NaOH was dissolved in distilled water flowed by the addition of 20 g BT nanoparticles, the mixture was stirred vigorously for 20 min. FeSO_4_·7H_2_O and FeCl_3_·6H_2_O were dissolved into 50 mL distilled water, respectively, then mixed together according to the mole ratio (Fe^3+^:Fe^2+^ = 2:1) and trickled into aqueous solution under vigorous stirring for 30 min at 40 °C. The pH of the aqueous solution was fixed at 11. The BT–Fe_3_O_4_ suspension was obtained which was washed with distilled water until the pH reached 7. Finally, BT–Fe_3_O_4_ hybrid particles were obtained by drying the suspension in a vacuum oven at 50 °C for 24 h.

### Preparation of BT–Fe_3_O_4_/PVDF nanocomposites

The BT–Fe_3_O_4_ fillers and PVDF matrix were mixed thoroughly with a torque rheometer for 30 min at 180 °C and then molded by hot pressing at approximately 180 °C and 15 MPa for 15 min to generate BT–Fe_3_O_4_/PVDF composites with a thickness of ≈200 μm and diameter of 4 cm. BT–Fe_3_O_4_/PVDF composites with different volume fractions of BT–Fe_3_O_4_ fillers were treated under a constant magnetic field with a magnetic induction density of 1.0 T for 30 min at 60 °C; henceforth, the composites treated by magnetic field will be referred to as BT–Fe_3_O_4_/PVDF#.

### Characterization

The phase compositions of the BT–Fe_3_O_4_ hybrid particles and the PVDF composites were analyzed using X-ray diffraction (XRD, Empyrean) using Cu Kα radiation at 40 kV and 40 mA. The microstructure of the PVDF composites was determined using SEM (Hitachi S-3400N) and the BT–Fe_3_O_4_ hybrid particles were analyzed using TEM (JEOL JEM-2100F). Prior to performing dielectric measurements, a thin layer of Al paste (diameter of 25 mm) was applied to the sides of the composites. The dielectric properties of the PVDF composites were determined in the frequency range of 100 Hz to 1 MHz at room temperature using a broadband dielectric spectral instrument (Novocontrol Alpha-A).

## Additional Information

**How to cite this article**: Zhang, C. *et al*. Enhanced dielectric properties of poly(vinylidene fluoride) composites filled with nano iron oxide-deposited barium titanate hybrid particles. *Sci. Rep.* **6**, 33508; doi: 10.1038/srep33508 (2016).

## Figures and Tables

**Table 1:** Comparison of the dielectric properties of our composites and reported literature materials at 100 Hz and room temperature.

Composites	*ε*	tan δ	*σ* (S/cm)	*f* (vol.%)	Ref.
BT@Fe_3_O_4_/PVDF	138	0.24	1.4 × 10^−9^	40	Our work
BT@Fe_3_O_4_/PVDF#	385	0.3	4.12 × 10^−9^	33	Our work
BT@Fe_3_O_4_/PVDF#-1 kHz	280	0.27	2.50 × 10^−8^	33	Our work
Hydantoin/BT/P(VDF-HFP)	48.3	0.06		50	[12]
BT/PVDF treated by PVP	115	0.02		60	[27]
BT–Ag/PVDF-1 kHz	160	0.11	9 × 10^−8^	56.8	[17]
BT/SiC/PVDF	213.8	0.27	3.31 × 10^−11^	35	[28]
BT@SnO_2_/PVDF	≈160	≈1.35	≈1 × 10^−8^	45	[29]
R-ZnO/BT/PVDF	175	0.45		30	[30]
Ag@TiO_2_/PTFE	240	1	1 × 10^−9^	70	[31]
Al/*β*-SiC_w_/PVDF	350	0.48		52	[32]
